# The hidden rhythms of epilepsy: exploring biological clocks and epileptic seizure dynamics

**DOI:** 10.1186/s42494-024-00197-w

**Published:** 2025-01-03

**Authors:** Ruili Niu, Xuan Guo, Jiaoyang Wang, Xiaofeng Yang

**Affiliations:** 1 Guangzhou National Laboratory, Guangzhou, 510005 China; 2 Department of Neurology, The First Affiliated Hospital, Guangzhou Medical University, Guangzhou, 510120 China; 3 Guangzhou Medical University, Guangzhou, 511436 China

**Keywords:** Epilepsy, Biological rhythm, Circadian genes, Chronotherapy, Neuromodulation

## Abstract

Epilepsy, characterized by recurrent seizures, is influenced by biological rhythms, such as circadian, seasonal, and menstrual cycles. These rhythms affect the frequency, severity, and timing of seizures, although the precise mechanisms underlying these associations remain unclear. This review examines the role of biological clocks, particularly the core circadian genes *Bmal1*, *Clock*, *Per*, and *Cry*, in regulating neuronal excitability and epilepsy susceptibility. We explore how the sleep-wake cycle, particularly non-rapid eye movement sleep, increases the risk of seizures, and discuss the circadian modulation of neurotransmitters like gamma-aminobutyric acid and glutamate. We explore clinical implications, including chronotherapy which refers to the practice of timing medical treatments to align with the body's natural biological rhythms, such as the circadian rhythm. Chronotherapy aligns anti-seizure medication administration with biological rhythms. We also discuss rhythm-based neuromodulation strategies, such as adaptive deep brain stimulation, which may dynamically change stimulation in response to predicted seizures in patients, provide additional therapeutic options. This review emphasizes the potential of integrating biological rhythm analysis into personalized epilepsy management, offering novel approaches to optimize treatment and improve patient outcomes. Future research should focus on understanding individual variability in seizure rhythms and harnessing technological innovations to enhance seizure prediction, precision treatment, and long-term management.

## Background

Epilepsy is a chronic neurological disorder characterized by recurrent, unprovoked seizures. It affects approximately 70 million people globally, making it one of the most common neurological diseases worldwide [1]. Beyond seizure episodes, epilepsy has far-reaching effects on patients’ quality of life, often contributing to cognitive decline, psychiatric comorbidities, social stigmatization, and increased mortality [2–4]. Despite the availability of anti-seizure medications (ASMs), about one-third of patients experience drug-resistant epilepsy, where seizures remain uncontrolled [5]. Understanding the mechanisms underlying epilepsy is critical for developing new therapeutic strategies and improving clinical outcomes.

Recent research highlights that biological rhythms-circadian (24-h cycles), ultradian (less than 24 h), and infradian (more than 24 h) are fundamental to the functioning of living organisms [6]. Among these, circadian rhythms are most well understood and are primarily regulated by the body's internal clock located in the suprachiasmatic nucleus (SCN) of the hypothalamus [7, 8]. This clock coordinates a wide range of physiological processes, including sleep-wake cycles, hormone secretion, metabolism, and neural activity [9–12]. Emerging evidence underscores the importance of these rhythms in brain function and neurological disorders, particularly epilepsy [13, 14].

The relationship between epilepsy and biological rhythms has been observed for centuries. Historical records from ancient civilizations, such as the Babylonians, noted connection between seizures and the lunar cycle [15]. In modern times, clinical observations confirm that seizures often follow specific rhythmic patterns, with circadian rhythms being particularly influential. Factors such as the sleep-wake cycle, light exposure, hormonal fluctuations, and even seasonal changes can affect seizure frequency, severity, and timing. Despite these observations, the mechanisms by which biological rhythms influence epileptogenesis and seizure susceptibility remain elusive [16–24].

This review aims to provide a comprehensive exploration of the complex relationship between biological rhythms and epilepsy. It will examine how various temporal rhythms-circadian cycles, sleep-wake patterns, hormonal fluctuations, and seasonal variations-affect neuronal excitability and seizure generation. By investigating the molecular and neurobiological pathways linking biological rhythms to epileptic activity, we will address current challenges in this emerging field and propose potential future research directions. Understanding these mechanisms may reveal novel insights into epilepsy management and open the door to chronotherapy strategies and other rhythm-based interventions or optimizing treatment outcomes.

## The rhythmic phenomena of epileptic seizures

### Circadian rhythms and epileptic seizures

Epileptic seizures often exhibit circadian patterns, with different types of seizures occurring more frequently at specific times of the day. Circadian rhythms, regulated by the body's internal clock located in the SCN, influence many physiological processes, including neuronal excitability and the sleep-wake cycle. For instance, studies by Durazzo et al have demonstrated that generalized tonic-clonic seizures often occur during the late night and early morning hours, which may be linked to circadian changes in cortical excitability and sleep architecture [25]. Frontal and parietal lobe seizures seem most likely to occur nocturnally, whereas occipital and temporal lobe seizures seem to have strong afternoon preferences [26]. A study by Quigg et al. confirmed that focal seizures follow this pattern, further supporting the influence of circadian-driven changes in brain physiology [27]. The relationship between sleep and epilepsy is also well-documented, with investigations revealing that non-rapid eye movement (NREM) sleep, characterized by slow-wave activity, is associated with an increased likelihood of seizure onset [28, 29, 30]. Bazil et al. demonstrated that slow-wave activity during non-rapid eye movement (NREM) sleep is closely related to seizure onset, particularly as increased slow-wave activity in this stage may lead to heightened cortical excitability, thus increasing the risk of seizures [31]. These studies collectively highlight the impact of circadian rhythms on the temporal dynamics of epileptic seizures.

### Seasonal variations and epileptic seizures

Seasonal variations also have a significant impact on the frequency of epileptic seizures, with multiple studies documenting increased seizure activity during specific seasons. For instance, Tomasović et al. found that seizure frequency rises during winter, particularly in December and January, and then declines in spring, suggesting a seasonal pattern in seizure occurrence [32]. This finding is consistent with research by Hammen et al., who also observed that winter months were associated with higher seizure frequencies, possibly due to the combined effects of reduced daylight hours and colder weather, which can influence both circadian rhythms and stress responses [33]. Furthermore, light exposure is another critical factor influencing seizure occurrence. Research indicates that seasonal changes in daylight duration may regulate seizure timing through the SCN, which controls melatonin and cortisol secretion. For example, Hammen et al. showed that specific weather conditions could act as triggers for seizures, likely due to environmental and circadian influences on the SCN [33]. In patients with photosensitive epilepsy, light stimulation can directly trigger seizures, underscoring the role of environmental light in modulating seizures activity [34]. AlDajani et al. proposed that the beneficial effects of light in reducing seizure frequency in epilepsy patients [35].

### Menstrual cycle and epileptic seizures: rhythms in women’s epilepsy

In women, epileptic seizures often follow a rhythmic pattern linked to hormonal fluctuations throughout the menstrual cycle, a phenomenon known as catamenial epilepsy. The influence of sex hormones, particularly estrogen and progesterone, plays a crucial role in modulating neuronal excitability. Estrogen tends to have a proconvulsant effect by increasing neuronal excitability, while progesterone is anticonvulsant due to its enhancement of GABAergic inhibition. Research conducted by Herzog et al. demonstrated that progesterone therapy could significantly reduce seizure frequency in women with catamenial epilepsy, indicating the therapeutic potential of hormonal modulation [36]. Similarly, some reports highlighted the anticonvulsant properties of progesterone metabolites, suggesting their applicability in reducing seizure susceptibility [37]. Women with catamenial epilepsy often experience an increase in seizure frequency during the perimenstrual phase, when estrogen levels rise and progesterone levels fall [36]. Dupont noted in one study that progesterone therapy may be effective for women with seizures associated with menstruation [38].

### Weekly and multiday cycles in epileptic seizures

In addition to circadian and menstrual rhythms, epileptic seizures can follow longer rhythmic cycles, such as weekly or multiday patterns. Approximately 60% of epilepsy patients report seizure clustering that follows a pattern of recurring every 7, 15, or 30 days [27, 39, 40]. This suggests the presence of supra-circadian rhythms that may be driven by biological cycles or external environmental factors. One of the hypothesized mechanisms for such patterns is the periodic fluctuation of stress hormones such as cortisol, which follows both circadian and ultradian rhythms. Elevated cortisol levels are known to lower seizure thresholds, and fluctuations in these levels may contribute to periodic increases in seizure activity [41]. While weekly seizure rhythms are less well understood compared to circadian and menstrual patterns, studies using long-term electroencephalogram (EEG) and seizure tracking devices are beginning to uncover the complex temporal dynamics that govern seizure timing [40].

## Molecular mechanisms: how biological rhythms affect epilepsy

### Role of clock genes in epilepsy

Two mechanisms have been proposed to elucidate the impact of the circadian clock on seizures: one suggests that canonical clock genes, such as *Bmal1* (basic helix-loop-helix Arnt like 1) and *Clock *(clock circadian regulator), directly contribute to epilepsy, while the other posits that the circadian clock operates through specific signaling pathways to influence epilepsy [42]. The canonical clock genes, such as *Bmal1*, *Clock*, *Per *(period circadian regulator), and *Cry *(circadian regulator cryptochrome) play essential roles in regulating circadian rhythms, which in turn modulate neuronal excitability and influence the likelihood of epileptic seizures [42]. These genes function in feedback loops, orchestrating the rhythmic expression of downstream targets involved in metabolism, hormone release, and neural activity [43–45]. In various animal models of epilepsy, the circadian oscillation and steady-state levels of circadian genes, including *Bmal1*, *Clock*, *Per1*, *Rev-Erbα*, and *Rorα*, have been found to be associated with epilepsy [44, 45]. Specifically, *Bmal1* and *Clock* form a heterodimer that drives the expression of *Per* and *Cry*, which in turn inhibit their own transcription by negatively regulating *Bmal1*-*Clock* activity, thereby maintaining a 24-h cycle [24, 43, 46, 47]. Further studies have expanded our understanding of how circadian clock components influence epilepsy. A study by Toh et al. found that *Per2* phosphorylation site mutation in familial advanced sleep phase syndrome disrupted the normal circadian rhythms of mice and increased their susceptibility to seizures. This highlights the role of the molecular clock in regulating neuronal excitability and seizure thresholds. The authors proposed that alterations in the circadian timing system might contribute to the development of epilepsy by disrupting the balance between excitatory and inhibitory neural activity. This finding supports the hypothesis that circadian disruption could play a role in modulating seizure onset and severity [48].

### Circadian regulation of GABA and glutamate

Neurotransmitters such as gamma-aminobutyric acid (GABA) and glutamate play crucial roles in maintaining the balance between neuronal excitation and inhibition, and their activity is tightly regulated by circadian rhythms. GABA, the primary inhibitory neurotransmitter, exhibits circadian variations in both its synthesis and release, with increased GABA-ergic tone observed during sleep, particularly in the NREM phase, when the risk of seizures is typically lower. Conversely, glutamate, the major excitatory neurotransmitter, shows increased activity during wakefulness and periods of heightened neural activity, corresponding with times of greater seizure risk. Liang et al. utilized a lithium-pilocarpine model to induce epilepsy in mice and simulated circadian disturbances by creating lesions in the SCN and specifically knocking out the *Bmal1* gene in the SCN neurons. SCN lesions intensified seizure activity, concomitant with hippocampal neuronal damage and GABAergic signaling impairment. This study underscores the crucial role of the SCN in modulating circadian rhythms and GABAergic function in the hippocampus, aggravating the severity of seizures [49]. Research has found that PAR bZip-deficient mice exhibited reduced expression of *Pdxk*, a gene encoding pyridoxal kinase [50]. This enzyme converts vitamin B6 derivatives into pyridoxal phosphate (PLP), the coenzyme of many enzymes involved in amino acid and neurotransmitter metabolism, such as glutamate decarboxylase (GAD) [51]. GAD catalyzes the conversion of glutamic to GABA, playing a vital role in maintaining GABA homeostasis in the brain. Downregulation of PLP has been linked to the increased susceptibility to seizures, highlighting a pathway relevant to epilepsy [44]. Additionally, Rev-Erbα has been identified as a potential epileptogenic factor and a primary source of seizure rhythmicity. Rev-erbα drives the expressions of GABA transporters (Slc6a1 and Slc6a11) via transcriptional repression of E4bp4, a negative regulator of these transporters. This enhances GABA reuptake, alleviating GABA-mediated inhibition and promoting epileptic seizures [52].

Glutamate, the major excitatory neurotransmitter, is also influenced by circadian rhythms. Studies have shown that the expression of glutamate receptors, particularly AMPA and NMDA receptors, varies throughout the day [53, 54]. Sandhu et al. observed significant 24-h oscillations of extracellular glutamate in the epileptogenic hippocampus, but not in the hippocampus of control animals. They speculate that the oscillations contribute to the rhythmicity of seizures in mesial temporal lobe epilepsy (MTLE) [55].

### Circadian regulation of inflammation and oxidative stress

Inflammation and oxidative stress could contribute to neuronal cell damage, increasing the risk of epileptic seizures [56]. Cells with mutations in circadian genes exhibit differential response to oxidative stress, indicating the involvement of circadian genes in regulating redox state and response to oxidative stress in a cell [57]. Studies demonstrate that core clock genes regulate the rhythmic expression of nuclear factor erythroid 2-related factor 2 (*Nrf2*) through E-box elements on the promoter in oxidative stress injury of multiple organs [58]. *Nrf2* is a regulator of cellular resistance to oxidants, controlling the expression of antioxidant response-dependent genes to manage the physiological and pathophysiological outcomes of oxidant exposure [59, 60]. Mice with *Bmal1* deletion experience oxidative stress and neuronal oxidative damage, resulting in neurodegeneration. The *Bmal1* complex with *Clock* or *Npas2* regulates cerebral redox homeostasis, connecting impaired clock gene function to neurodegeneration [61]. This represents another potential molecular pathogenic mechanism linking circadian rhythms to epilepsy.

## Sleep-wake cycle and epilepsy

### Role of NREM and REM sleep

Epileptic seizures display a well-documented relationship with sleep, particularly during NREM sleep. NREM sleep is characterized by slow-wave oscillations, which are known to facilitate the synchronization of neuronal networks [62]. Epileptic seizures exhibit a well-documented relationship with sleep, particularly during NREM sleep. NREM sleep is characterized by two distinct stages: Stage 2 and Stage 3, each of which plays a unique role in brain activity and seizure dynamics. Stage 2 sleep is characterized by sleep spindles, which are brief bursts of high-frequency oscillations (12–16 Hz in humans) that are thought to be involved in the synchronization of neuronal networks across cortical areas. This synchronization is generally protective, facilitating cortical inhibition that can reduce seizure susceptibility. However, abnormal spindle activity or disrupted synchronization can contribute to cortical hyperexcitability, which may increase the likelihood of seizure initiation [63]. Studies have shown that disruption in sleep spindles is associated with a reduced seizure threshold and enhanced seizure propagation in individuals with epilepsy. For example, Lee et al. demonstrated that alterations in spindle activity during NREM sleep may facilitate the generation of epileptic spasms through mechanisms that involve the thalamocortical circuit [64].

On the other hand, Stage 3 Sleep, or deep sleep, is characterized by slow-wave activity (SWA), particularly delta waves (0.5–4 Hz), which are thought to represent a high level of cortical synchronization. While slow-wave oscillations are generally considered to promote cortical inhibition and play a crucial role in seizure suppression, excessive or abnormal SWA during NREM sleep may contribute to hyperexcitability in the thalamocortical circuit, facilitating seizure onset [65]. Indeed, Lee et al. found that altered slow oscillations in the neocortex, which are prominent during Stage 3 sleep, can lead to the initiation of seizures, particularly when these oscillations become pathologically amplified [64]. Moreover, excessive SWA during deep sleep has been implicated in the propagation of seizures, especially in focal epilepsies, where abnormal synchronization in deep sleep stages can increase the risk of generalized seizures [66].

Therefore, although both Stage 2 and Stage 3 sleep contribute to the synchronization of neuronal networks, their effects on epilepsy can differ based on the specific oscillatory patterns present in each stage. Stage 2 sleep, with its characteristic sleep spindles, is more involved in cortical inhibition and network consolidation, and disruptions in this process may lead to seizure susceptibility. Stage 3 sleep, dominated by slow-wave activity, plays a dual role: while it typically helps suppress seizures, excessive slow-wave activity can shift the balance towards hyperexcitability, thus increasing seizure risk.

The slow oscillations observed during NREM sleep are particularly implicated in the generation of epileptic spasms [64, 66, 67]. Lee et al. investigated the role of neocortical slow oscillations in epilepsy using a tetrodotoxin (TTX) animal model and found that these oscillations are crucial for the onset of seizures. Their study also demonstrated that seizures originating from neocortical layers during NREM sleep share similarities with preictal pauses, which are related to NREM down states. This suggests that the same network mechanisms that govern normal sleep physiology are co-opted to generate pathological discharges in epilepsy [64].

Comparatively, REM sleep is associated with desynchronization of brain activity, which appears to be protective against seizure generation. Seizures are significantly less frequent during REM sleep, likely because of the more desynchronized and less synchronous neural activity observed during this sleep stage [67]. Thus, the contrasting roles of NREM and REM sleep underscore the complexity of sleep-related seizure dynamics.

### Sleep deprivation and seizure susceptibility

Sleep deprivation is a well-known trigger for seizures, particularly in individuals with epilepsy. The mechanism behind this increased seizure susceptibility lies in the disruption of the balance between excitatory and inhibitory signals in the brain. Studies have shown that sleep deprivation reduces GABAergic inhibition while increasing excitatory neurotransmitter levels, leading to a lower seizure threshold [68, 69].

Sleep deprivation disrupts circadian rhythms and increases seizure risk. This occurs because it alters neural network synchronization, promoting abnormal discharges that can trigger seizures. This is particularly concerning in patients with nocturnal seizures, who are more likely to experience seizures following periods of sleep loss. A study by Dell'Aquila and Soti highlighted that sleep deprivation significantly increases the risk of epileptic seizures by disrupting the synchrony of neural networks, which is critical in preventing abnormal discharges associated with seizure activity [68]. Moreover, Eide et al. demonstrated that sleep deprivation impairs the molecular clearance process in the brain, leading to an accumulation of metabolic waste products, which may destabilize neural circuits and increase the likelihood of seizures [69]. Additionally, Frauscher and Gotman explored how sleep deprivation affects the brain's oscillatory activity, emphasizing the disruption of normal sleep-related oscillations, which can trigger interictal discharges and seizures in patients with focal epilepsy [67]. These findings underline the complex relationship between sleep disturbances and seizure risk, particularly in those prone to nocturnal seizures.

### Shared neural circuits: sleep and epilepsy

The thalamocortical circuit is a key player in both the regulation of sleep and the generation of seizures. This circuit is involved in generating the sleep spindles and slow-wave oscillations observed during NREM sleep, and its hyperexcitable state during epilepsy can lead to seizure propagation. Studies have shown that changes in thalamocortical oscillations are linked to both normal sleep activity and epileptiform discharges, highlighting the shared neural pathways between sleep and seizure generation. For instance, Lee et al. found that altered thalamocortical oscillations, particularly in the slow-wave range, play a crucial role in both the regulation of sleep and the initiation of seizures in models of focal epilepsy [64]. Additionally, Sillanpää and Shinnar demonstrated that abnormal thalamocortical synchronization in the early stages of epilepsy was associated with disrupted sleep patterns, which further exacerbated seizure susceptibility [3]. These findings provide compelling evidence of the shared mechanisms between sleep regulation and seizure generation, emphasizing the importance of thalamocortical circuitry in both processes.

Additionally, sharp wave-ripple complexes (SPW-Rs) during NREM sleep are essential for memory consolidation, primarily through their coupling with cortical oscillations. However, when SPW-Rs become exaggerated and more synchronized, they can transition into pathological ripples, which are associated with epileptiform activity and seizure generation. While physiological ripples play a beneficial role in memory consolidation, pathological ripples represent a maladaptive form of synchronization that contributes to the generation and spread of seizures. The precise mechanisms by which physiological ripples transition into pathological ripples are still under investigation, but they are believed to involve alterations in hippocampal network stability and increased neuronal synchronization [67]. Additionally, Bernard et al. observed that in patients with mesial temporal lobe epilepsy, exaggerated SPW-Rs during sleep were linked to an increased probability of seizures in the subsequent wake periods [66]. The close link between sleep-related oscillations and epileptic discharges suggests that therapeutic interventions aimed at normalizing these oscillations could help reduce seizures in epilepsy. For instance, Lee et al. proposed that targeted modulation of SPW-Rs, such as through deep brain stimulation, could offer a potential approach to controlling seizure activity [64].

## Biological rhythms and sudden unexpected death in epilepsy (SUDEP)

### Circadian rhythms and the risk of SUDEP

SUDEP is a major concern for patients with epilepsy, and emerging evidence suggests that circadian rhythms may influence its occurrence. Circadian rhythms regulate numerous physiological processes, including heart rate variability, respiratory patterns, and autonomic function, all of which are known to play a role in SUDEP risk. Disruptions in these biological rhythms, particularly in heart and respiratory functions, are linked to an increased vulnerability to SUDEP during specific times of the day [16, 70–72].

Joyal et al. demonstrated the current state of understanding of the relationship between respiratory function, sleep state and time of day, and epilepsy. They highlight sleep as a particularly vulnerable period for individuals with epilepsy and press that this topic warrants further investigation in order to develop therapeutic interventions to mitigate the risk of SUDEP [73].

Cardiac rhythm disturbances, such as bradycardia, asystole, and arrhythmias, have been observed in epilepsy patients, especially during sleep and nighttime seizures. These disruptions are modulated by circadian influences on parasympathetic nervous system activity, which is typically dominant at night [74]. In patients with epilepsy, the autonomic nervous system may already be compromised, leading to an increased susceptibility to cardiac events following seizures.

Similarly, respiratory dysregulation, manifesting as central or obstructive apnea, has been observed in patients who succumb to SUDEP. Sleep-induced changes in respiratory control, particularly the body's reduced sensitivity to CO_2_ levels during NREM sleep, may worsen during seizures, increasing the risk of fatal outcomes. Thus, circadian rhythms influence both cardiac and respiratory systems, and their disruption during seizures can lead to catastrophic outcomes like SUDEP [73, 75].

### Specific time windows of increased SUDEP risk

Research has shown that SUDEP risk is not evenly distributed across the 24-h day but is concentrated during certain periods, notably at night and in the early morning hours. The majority of SUDEP cases occur during sleep, and more specifically, during the transition from NREM to REM sleep, when respiratory control is particularly vulnerable [76, 77]. Sleep-induced reductions in arousal thresholds and the body's reduced sensitivity to oxygen deprivation (hypoxia) and elevated carbon dioxide levels (hypercapnia) during sleep stages may contribute to the increased risk during this time [78].

Nocturnal seizures are particularly concerning, as they tend to coincide with these critical periods of reduced autonomic stability. Studies have suggested that SUDEP events are more likely to occur between midnight and 6 a.m., correlating with periods of heightened parasympathetic activity and reduced autonomic resilience [79]. These findings highlight the importance of considering not only seizure frequency but also the timing of seizures when assessing SUDEP risk [80].

Additionally, the sleep-wake cycle and its interaction with seizure timing may influence SUDEP risk. Seizures occurring during NREM sleep, which is associated with synchronized cortical activity, may more easily propagate through brain networks and disrupt autonomic functions. The circadian trough of sympathetic tone during the early morning may further diminish the body’s capacity to recover from seizure-induced autonomic disturbances [73].

## Clinical applications: time-based epilepsy therapies

### Chronotherapy in epilepsy treatment

Chronotherapy, the alignment of treatment schedules with biological rhythms, has been explored as a promising approach for optimizing epilepsy management. By adjusting the timing of drug administration to coincide with circadian peaks of seizure activity, chronotherapy aims to enhance drug efficacy while minimizing side effects [14, 42, 81].

The pharmacokinetics and pharmacodynamics of ASMs vary depending on the time of day due to circadian influences on drug absorption, metabolism, and elimination. For example, medications like carbamazepine and valproate are more effective when administered during periods of increased seizure susceptibility, typically in the late evening for nocturnal seizures or early morning for daytime seizures [82]. Tailoring ASMs regimens to each patient’s specific seizure pattern, as tracked through seizure diaries or long-term monitoring, can improve seizure control [42].

In addition, chronotherapy may reduce the side effects associated with ASMs by aligning drug peaks with circadian troughs in metabolic activity. For instance, administering ASMs during periods of low hepatic enzyme activity can reduce drug breakdown, maintaining therapeutic levels for longer and decreasing the need for high dosages [42, 83].

### Rhythm-based neuromodulation interventions

Neuromodulation, including vagus nerve stimulation (VNS) and deep brain stimulation (DBS), has emerged as a therapeutic option for patients with refractory epilepsy. Increasing evidence suggests that the timing of neuromodulatory interventions can be optimized by considering the patient’s biological rhythms [84, 85].

For example, DBS targeting the anterior nucleus of the thalamus (ANT) has shown the potential for controlling seizures more effectively when the stimulation is synchronized with the patient’s circadian rhythm of seizure activity. This approach, known as adaptive DBS, involves programming the stimulation to occur during the periods of highest seizure risk, such as during nighttime or early morning hours when seizures are more likely to occur [86]. Worrell and Kermen demonstrated that adaptive DBS, synchronized with circadian rhythms, could significantly reduce seizure frequency in patients, suggesting that circadian timing of stimulation may enhance its effectiveness [87]. Charlebois et al. reported that circadian modulation of baseline broadband activity is a biomarker of response to responsive nerve stimulator (RNS) early during therapy. This marker has the potential to identify patients who are likely to respond to mesial temporal RNS [19].

Similarly, VNS may be optimized by delivering stimulation in alignment with biological rhythms that influence seizure susceptibility. For instance, VNS programmed to deliver increased stimulation during periods of circadian vulnerability (e.g., late night or early morning) could provide more effective seizure control while reducing overall energy consumption and minimizing side effects [88, 89].

These rhythm-based neuromodulation interventions highlight the potential of aligning therapeutic strategies with the patient’s natural biological rhythms to enhance efficacy and provide more personalized treatment.

## Future directions and challenges

### Unresolved mysteries: unanswered questions in biological rhythms and epilepsy

Despite significant advancements in understanding the relationship between biological rhythms and epilepsy, several questions remain unresolved. One of the most perplexing mysteries is the individual variability in rhythmic seizure patterns. While some patients exhibit clear circadian or menstrual cycles in their seizures, others display highly irregular patterns, which makes it difficult to predict seizure timing [90]. This variability may be due to a combination of genetic, environmental, and lifestyle factors, but the precise mechanisms are still unclear [91].

Recent studies have highlighted the complexity of these individual differences. For instance, Ramgopal et al. found that even among patients with similar epilepsy syndromes, there can be marked variations in seizure chronotypes. Their analysis of 324 patients revealed that while 45% showed strong circadian patterns, others demonstrated multi-day cycles or apparently random distributions. These findings suggest that standard chronotherapy approaches may need significant individualization [92].

Another unresolved issue is the interplay between different biological rhythms, such as the circadian and menstrual cycles, in modulating seizure activity. Herzog et al. demonstrated in their cohort study of women that seizure frequency can vary by up to 300% depending on the phase alignment between circadian and menstrual cycles [93]. For example, the overlap between circadian and infradian rhythms in women with catamenial epilepsy complicates the predictability of seizure timing, and understanding the interaction between these cycles remains a key challenge. Future research should focus on disentangling these complex interactions to offer more personalized epilepsy management strategies.

Advances in artificial intelligence (AI) and data analytics have enabled complex modeling of epilepsy rhythms using multi-modal inputs, including genetic profiles, wearable biosensor data, and environmental factors. Machine learning algorithms now allow real-time seizure precursor detection, surpassing traditional methods in predictive accuracy. Recent studies have shown that machine learning techniques, such as deep learning and support vector machines (SVM), offer effective methods for predicting epileptic seizur**e**s based on EEG signals. Kerr et,al. highlighted the use of Explainable Artificial Intelligence (XAI) to develop efficient seizure detection models using EEG signals. They research underscores the potential for feature reduction and strategically selected electrodes to create more efficient and generalized models for seizure prediction [94]. Yu et, al. explored the use of wearable devices for seizure detection. In this study, custom-developed deep learning models were applied to signals from wrist- or ankle-worn devices to detect a broad range of seizure types. Their study demonstrated that accelerometry (ACC) and photoplethysmography (PPG) signals, when processed with a convolutional neural network (CNN) and a CNN-long short-term memory (LSTM) model, achieved a sensitivity of 83.9% and a false positive rate of 35.3%. This method detected 19 out of 28 seizure types with high performance, contributing to noninvasive, real-time seizure detection [95]. Karoly introduced a proof-of-concept for integrating circadian rhythm frameworks into seizure prediction models, offering new insights for improving these algorithms [96].

Recent advances in AI and data analytics have significantly enhanced seizure prediction models by integrating multi-modal inputs, such as genetic profiles, wearable biosensor data, and environmental factors. Machine learning techniques, including deep learning, SVM, and XAI, have demonstrated high efficacy in predicting seizures based on EEG and wearable device signals, offering promising noninvasive, real-time detection methods.

## Conclusions

### Summary of the complex relationship between epilepsy and biological rhythms

This review highlights the intricate and multifaceted relationship between biological rhythms and epilepsy. Circadian rhythms, sleep-wake cycles, and other temporal patterns such as menstrual and seasonal rhythms all play significant roles in modulating neuronal excitability and the likelihood of seizures. The molecular underpinnings of these effects involve key clock genes, such as *Bmal1* and *Clock*, which are critical in regulating both circadian rhythms and seizure susceptibility.

The effects of circadian dysregulation, sleep deprivation, and autonomic dysfunction on epilepsy are well-documented, particularly in relation to the increased risk of SUDEP. By synchronizing therapeutic interventions with these biological rhythms, it is possible to enhance treatment efficacy and reduce the risk of adverse outcomes.

The integration of chronobiology into epilepsy management represents a promising direction for precision medicine. Advances in computational modeling, combined with multi-modal biosensor data, are enabling the development of more sophisticated chronotherapeutic strategies. Recent progress in closed-loop neuromodulation systems shows potential for rhythm-responsive interventions that adjust stimulation parameters based on individual circadian profiles and real-time physiological markers.

Looking ahead, AI-driven systems will become more integrated, synthesizing multiple data streams in real-time to provide actionable insights for both clinicians and patients. The ultimate goal is to develop predictive models capable of accurately forecasting seizure risk, enabling preventive interventions while accounting for individual circadian rhythms and environmental factors. This precision medicine approach grounded in chronobiology may significantly reduce seizure burden and improve patient outcomes.

## Data Availability

Not applicable.

## Abbreviations

ACC: Accelerometry
AI: Artificial intelligence
ANT: Anterior nucleus of the thalamus
ASMs: Anti-seizure medications
CNN: Convolutional neural network
EEG: Electroencephalogram
GABA: Gamma-aminobutyric acid
GAD: Glutamate decarboxylase
LSTM: Long short-term memory
MTLE: Mesial temporal lobe epilepsy
NREM: Non-rapid eye movement
Nrf2: Nuclear factor erythroid 2-related factor 2
PLP: Pyridoxal phosphate
PPG: Photoplethysmography
REM: Rapid eye movement
SCN: Suprachiasmatic nucleus
SPW-Rs: Sharp wave-ripple complexes
SUDEP: Sudden unexpected death in epilepsy
SVM: Support vector machines
TTX: Tetrodotoxin
VNS: Vagus nerve stimulation
XAI: Explainable artificial intelligence
